# Uptake and Glycosylation of Smoke-Derived Volatile Phenols by Cabernet Sauvignon Grapes and Their Subsequent Fate during Winemaking

**DOI:** 10.3390/molecules25163720

**Published:** 2020-08-14

**Authors:** Colleen Szeto, Renata Ristic, Dimitra Capone, Carolyn Puglisi, Vinay Pagay, Julie Culbert, WenWen Jiang, Markus Herderich, Jonathan Tuke, Kerry Wilkinson

**Affiliations:** 1 School of Agriculture, Food and Wine, The University of Adelaide, PMB 1, Glen Osmond, SA 5064, Australia; colleen.szeto@adelaide.edu.au (C.S.); renata.ristic@adelaide.edu.au (R.R.); dimitra.capone@adelaide.edu.au (D.C.); carolyn.puglisi@adelaide.edu.au (C.P.); vinay.pagay@adelaide.edu.au (V.P.); markus.herderich@awri.com.au (M.H.); 2 The Australian Research Council Training Centre for Innovative Wine Production, PMB 1, Glen Osmond, SA 5064, Australia; 3 The Australian Wine Research Institute, PO Box 197, Glen Osmond, SA 5064, Australia; julie.culbert@awri.com.au (J.C.); maddy.jiang@awri.com.au (W.J.); 4 School of Mathematical Sciences, The University of Adelaide, SA 5000, Australia; simon.tuke@adelaide.edu.au

**Keywords:** acid hydrolysis, cresols, guaiacol, particulate matter, rate-all-that-apply, sensors, smoke taint, syringol, volatile phenol glycosides, wine

## Abstract

Wine made from grapes exposed to bushfire smoke can exhibit unpleasant smoky, ashy characters, which have been attributed to the presence of smoke-derived volatile phenols, in free or glycosylated forms. Here we report the uptake and glycosylation of volatile phenols by grapes following exposure of Cabernet Sauvignon vines to smoke, and their fate during winemaking. A significant delay was observed in the conversion of volatile phenols to their corresponding glycoconjugates, which suggests sequestration, the presence of intermediates within the glycosylation pathway and/or other volatile phenol storage forms. This finding has implications for industry in terms of detecting smoke-affected grapes following vineyard smoke exposure. The potential for an in-canopy sprinkler system to mitigate the uptake of smoke-derived volatile phenols by grapes, by spraying grapevines with water during smoke exposure, was also evaluated. While “misting” appeared to partially mitigate the uptake of volatile phenols by grapes during grapevine exposure to smoke, it did not readily influence the concentration of volatile phenols or the sensory perception of smoke taint in wine. Commercial sensors were used to monitor the concentration of smoke particulate matter (PM) during grapevine exposure to low and high density smoke. Similar PM profiles were observed, irrespective of smoke density, such that PM concentrations did not reflect the extent of smoke exposure by grapes or risk of taint in wine. The sensors could nevertheless be used to monitor the presence of smoke in vineyards during bushfires, and hence, the need for compositional analysis of grapes to quantify smoke taint marker compounds.

## 1. Introduction

“Smoke taint” describes unpleasant smoky, medicinal and ashy characters that can arise in wine following grapevine exposure to bushfire smoke [1,2]. The intensity of smoke taint depends on the timing and duration of smoke exposure [3,4], grape variety [5], fruit maturity at harvest [6] and winemaking practices (e.g., skin contact time during fermentation) [7,8]. The presence of smoke taint can be determined in wine by sensory analysis and/or by measuring the concentrations of smoke-derived volatile phenols, including guaiacol, 4-methylguaiacol, *o-*, *m-* and *p*-cresol, syringol and 4-methylsyringol [5,9,10,11]; but in grapes, volatile phenols accumulate in glycoconjugate forms (i.e., as mono-, di- and even trisaccharides [12,13,14,15,16,17]), complicating their detection. Analytical methods have been developed to measure volatile phenol glycoconjugates, either directly by liquid chromatography-tandem mass spectrometry [11,18], or indirectly, by quantifying the volatile phenols released following acid or enzyme hydrolysis [19,20,21]. However, to date, few studies have monitored temporal changes in grape volatile phenol glycoconjugates following grapevine exposure to smoke or the fate of volatile phenol glycoconjugates during winemaking. 

Dungey and coworkers reported the accumulation of guaiacol glycoconjugates in smoke-affected Merlot and Viognier grapes, and while glycoconjugates were detected 3–5 days post-smoke exposure, significant increases were observed in grapes sampled 12 or more days after smoke exposure, leading the authors to conclude that glycosylation occurred over 10 to 14 days [18]. In a more recent study, van der Hulst and colleagues found low levels of volatile phenols (≤4 µg/L) in grapes sampled one day after grapevine exposure to smoke, but the concentrations of key volatile phenol glycoconjugates increased significantly between 1 and 7 days post-smoke exposure [16]. Unfortunately, neither of these studies measured grape volatile phenols immediately after smoke exposure, nor did they involve winemaking, so questions remain regarding the uptake of smoke-derived volatile phenols by grapes and how glycoconjugate profiles change as grapes are processed into wine. This study sought to address these knowledge gaps by measuring grape volatile phenols, in free and glycosylated forms, following grapevine exposure to low and high density smoke, and then in corresponding wines. The study also included preliminary evaluations of: (i) in-canopy misting as a strategy for mitigating the uptake of volatile phenols during grapevine smoke exposure; and (ii) a commercial sensor for monitoring vineyard exposure to smoke during a bushfire. 

A recent study used in-canopy sprinklers to mitigate the effects of heat stress in Cabernet Sauvignon berries during ripening, by spraying water within the bunch zone (for 20 s/10 min when air temperature exceeded 38 °C) to cool the vine microclimate by 3–5 °C [22]. Attempts to “wash” grapevines/fruit following exposure to smoke (using water, 5% aqueous ethanol or milk) did not reduce the guaiacol concentration of grapes or juice [23,24], which might reflect rapid diffusion of smoke-derived volatile phenols into berries. The in-canopy sprinkler system could instead be used to “wash” grape bunches during smoke exposure, i.e., to potentially mitigate the uptake of smoke-derived volatile compounds through the removal of smoke particles in a manner similar to the way in which rain cleanses the atmosphere by capturing aerosols [25]. This study therefore included an investigation into the impact of in-canopy misting during grapevine exposure to smoke on the concentration of smoke taint marker compounds in grapes and wine, and the perception of smoke taint in wine. Additionally, commercial sensors were deployed during field trials to measure the concentration of particulate matter, in order to determine their suitability for monitoring smoke from bushfires, with different densities of smoke achieved by burning different amounts of fuel. 

## 2. Results and Discussion

The composition of grapes from control and smoke-exposed grapevines were determined just prior to smoke exposure (i.e., at t = 0, being approximately 7 days post-veraison); then, at 1 h, 1 day and 1 week post-smoke exposure (hereafter t = 1, t = 2 and t = 3, respectively), and again at harvest/maturity (hereafter t = 4, being 4 weeks post-smoke exposure). The composition and sensory profiles of control and smoke-affected wines were also determined. This enabled investigation of: (i) the uptake and in vivo glycosylation of smoke-derived volatile phenols by grapes; and (ii) the subsequent fate of volatile phenols (and their glycoconjugates) during winemaking.

### 2.1. Uptake and Glycosylation of Smoke-Derived Volatile Phenols by Grapes

Prior to smoke exposure (i.e., at t = 0), grape juice volatile phenol concentrations were ≤3.6 µg/L, with the exception of syringol which ranged from 6.2 to 12 µg/L (Table 1). Elevated volatile phenol levels were detected in juice from grapes sampled 1 h after exposure to smoke (i.e., at t = 1), irrespective of smoke density, albeit only phenol and cresol concentrations of grapes exposed to low density smoke (“LS”) were significantly different (*P* = 0.034 and 0.033, respectively) from their corresponding control grapes (i.e., “C” at t = 1). Guaiacol and syringol were detected at the highest concentrations, being 108 and 126 µg/L in juice from grapes exposed to high density smoke (“HS”) respectively, followed by cresols, phenol, 4-methylguaiacol and 4-methylsyringol, which were detected at 83, 55, 20 and 17 µg/L respectively (Table 1). Within 24 h of smoke exposure, the elevated volatile phenol levels observed in LS and HS grapes had decreased by as much as 75%, such that syringol, 4-methylguaiacol, phenol and 4-methylsyringol levels were not significantly different from those detected in control grapes (Table 1). Guaiacol and cresol concentrations similarly decreased, but remained significantly higher in HS grapes (than in control grapes) until one and four weeks after smoke exposure (i.e., until t = 3 and t = 4), respectively (Table 1). 

These results demonstrate the rapid uptake of volatile phenols from smoke by grapes during grapevine exposure to smoke, and as reported in previous studies [12,13,14,15,16,17,18], their subsequent in vivo glycosylation. However, while some volatile phenol glycoconjugates (measured as syringol glucose-glucoside equivalents) were observed at significantly elevated concentrations in HS grapes 24 h after smoke exposure (i.e., at t = 2), namely, syringol glucose glucoside (gentiobioside), cresol glucoside and cresol rutinoside (Table S1), accumulation of other volatile phenol glycoconjugates seemingly occurred one to four weeks after smoke exposure (i.e., between t = 3 and t = 4) (Table S1). By harvest (i.e., at t = 4), the concentrations of guaiacol pentose glucoside, phenol pentose glucoside, cresol pentose glucoside and syringol glucose glucoside in HS grapes were 803, 576, 988 and 535 µg/kg respectively (Table S1); meanwhile, pentose glucosides of 4-methylguaiacol and syringol, 4-methylsyringol glucose glucoside, and rutinosides of 4-methylguaiacol, phenol and cresols ranged from 98 to 258 µg/kg (Table S2). Other volatile phenol glycoconjugates, including glucosides, were detected at ≤50 µg/kg.

The glycoconjugate profiles observed for LS and HS grapes were similar to those reported in previous studies involving the application of smoke to grapevines of different varieties [11,13,16], in that pentose glucosides of guaiacol, 4-methylguaiacol, cresol, phenol and syringol, and glucose glucosides (gentiobiosides) of syringol and 4-methylsyringol were most abundant in smoke-exposed grapes (at harvest). In the latter study, van der Hulst and colleagues reported a similar (albeit shorter) delay in the accumulation of volatile phenol glycoconjugates. Volatile phenols were detected at ≤4 µg/L in grapes sampled 1 d after grapevine exposure to smoke, but the concentrations of several volatile phenol glycoconjugates increased significantly between one and seven days post-smoke exposure, especially in Merlot vines; glycoconjugate levels then remained relatively constant until harvest [16].

The apparent delay between the “disappearance” of volatile phenols and “appearance” of their glycoconjugates might be explained by sequestration of volatile phenols in plant cell walls or vacuoles, the presence of “intermediates” within the glycosylation pathway and/or other volatile phenol metabolites, as suggested by Noestheden and colleagues [24]. Nevertheless, this delay has important implications for the wine industry, since it suggests that analysis of volatile phenols and/or their glycoconjugates in grapes sampled between one and seven days after smoke exposure (and possibly longer in some grape varieties), might underestimate the levels that are subsequently detected in mature grapes and/or wine, i.e., there is potential for the level of smoke taint to be underestimated.

**Figure 1:**
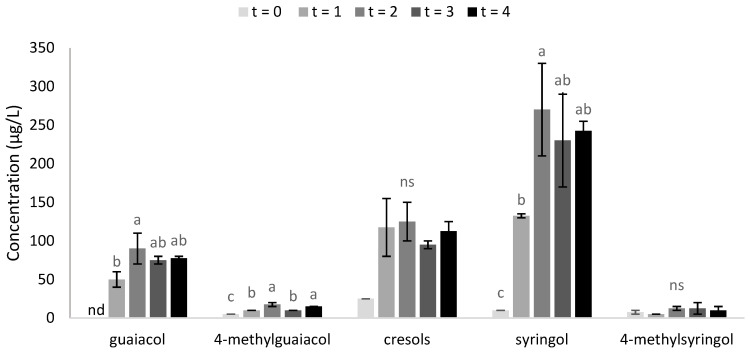
Volatile phenol concentrations in acid hydrolysates derived from HS grapes sampled at different time points, i.e., immediately prior to smoke exposure (t = 0); 1 h after smoke exposure (t = 1); 1 day after smoke exposure (t = 2); 7 days after smoke exposure (t = 3); and 4 weeks after smoke exposure (t = 4) being maturity. Values are means of two replicates (*n* = 2) ± standard errors. Different letters indicate statistical significance (*P* = 0.05, one-way ANOVA); ns = not significant; nd = not detected.

Several previous studies employed enzyme, acid and/or base hydrolysis of grape homogenate, juice or wine to facilitate quantification of glycoconjugate forms of volatile phenols [1,15,19,20,21,24,26]. Noestheden and colleagues optimized a method for measuring glycosidically-bound volatile phenols in smoke-exposed grapes using acid-mediated hydrolysis [15], key recommendations being the use of Strata X solid phase extraction (SPE) cartridges for isolation of glycoconjugates and PTFE tubes for acid digestion (instead of borosilicate glass vials, which seem to interfere with the assay yielding low recoveries for some volatile phenols). In the current study, a similar method was applied to HS grapes (at each time point) to further investigate the accumulation of volatile phenols in bound forms (Figure 1). The acid hydrolysate from HS grapes sampled at t = 0 contained low levels of volatile phenols (i.e., 0–25 µg/L; Figure 1). Acid hydrolysis of smoke-affected grape samples liberated ≤13 µg/L of 4-methylguaiacol and 4-methylsyringol, irrespective of sampling time, but released 50–90, 70–100 and 120–260 µg/L of guaiacol, cresols and syringol, respectively (Figure 1). These results were consistent with: 4-methylguaiacol and 4-methylsyringol being the least abundant volatile phenols in HS grapes, especially in free form at t = 1 (Table 1) and in glycosylated forms at t = 4 (Table 1 and Table S1); and guaiacol, cresols and syringol being the most abundant volatile phenols, in both free and glycosylated forms (Table 1 and Table S1). 

The elevated concentrations of guaiacol, cresols and syringol in the t = 1 hydrolysate (compared with the t = 0 hydrolysate) suggest these compounds were being metabolized within the berry during and/or immediately after smoke exposure. The significant increase in guaiacol and syringol concentrations observed for hydrolysates between t = 1 and t = 2 might reflect conversion of free forms of these volatile phenols into bound forms. However, the glycoconjugate levels at t = 2 do not account for the quantities of guaiacol and syringol observed in t = 2 hydrolysate, suggesting the presence of intermediates (or other metabolites) of these volatile phenols. The guaiacol and syringol levels in t = 3 and t = 4 hydrolysates were not significantly different from those in either t = 1 or t = 2 hydrolysates; surprisingly, there was also no statistical significance amongst cresol concentrations in acid hydrolysates, at any time point (Figure 1). These results are particularly interesting given that by t = 4, substantial quantities of guaiacol, cresol and syringol glycoconjugates had accumulated in HS grapes (Table 1 and Table S1) and high performance liquid chromatography-tandem mass spectrometry (HPLC–MS/MS) analysis of hydrolysates confirmed there were no volatile phenol glycoconjugates remaining after acid hydrolysis (data not shown). Based on the concentrations of guaiacol and cresol pentose glucosides and syringol glucose glucoside found in mature HS grapes (i.e., the most abundant glycoconjugates for each of these volatile phenols), complete hydrolysis would be expected to yield guaiacol, cresol and syringol concentrations of at least 238, 265 and 172 µg/L, respectively; i.e., mass balance could not be achieved. Whereas Noestheden and colleagues reported quantitative recovery of free volatile phenols following acid hydrolysis of their corresponding glucosides [21], similar results were not obtained in the current study, for the glycoconjugates measured. 

The acid hydrolysis experiments performed as part of the current study are considered preliminary only. More detailed studies could not be pursued because very limited quantities of samples remained after completion of other analyses, but will instead be undertaken as part of ongoing smoke taint research. Nevertheless, these findings further support the existence of other intermediates and/or storage forms of volatile phenols. They also demonstrate the potential for acid hydrolysis to be used to further investigate the uptake and accumulation of smoke-derived volatile phenols by grapes, and to detect smoke taint in grapes, especially when direct analysis of volatile phenols and/or their glycoconjugates might not account for the presence of all forms of volatile phenols (e.g., in the days immediately after smoke exposure). 

### 2.2. Comparison of Smoke Taint Markers in Grapes vs. Wine

The chemical analyses performed in the current study enabled comparisons to be made between the compositions of control and smoke-exposed grapes, and their corresponding wines, to determine the fate of volatile phenols (both free and glycosylated forms) during winemaking.

Prior to smoke exposure, the background (“natural”) levels of volatile phenols present in LS and HS grapes were ≤7.8 µg/L (Table 1), with syringol being the most abundant volatile phenol. After smoke exposure (i.e., at t = 1), guaiacol and syringol were the most abundant volatile phenols (in both LS and HS grapes), followed by cresols, phenol, 4-methylguaiacol and 4-methylsyringol. However, by maturity, there were no significant differences in the syringol or 4-methylsyringol levels of C, LS and HS grapes (Table 1). Elevated levels of guaiacol, phenol and cresols in HS grapes (and of phenol and cresols in LS grapes, but to a lesser extent) provided the only evidence of smoke exposure at maturity (i.e., at t = 4). As outlined above, this was because volatile phenols were predominantly present in conjugate forms. In LS and HS wines, guaiacol was the most abundant volatile phenol, albeit combined, cresols were present at similar levels (Table 1); syringol, 4-methylguaiacol and 4-methylsyringol concentrations were ≤5 µg/L (and phenol was not measured in wines). These results show good agreement with compositional analyses of Cabernet Sauvignon grapes and/or wines reported in several previous studies on smoke taint [5,20,26].

Several studies have shown that a significant pool of volatile phenol glycoconjugates remains in wine after fermentation [5,6,7,17,19,20,27,28]. However, to date, the changes in glycoconjugate concentrations during fermentation have not been extensively studied. In this study, the relative abundance of volatile phenol glycoconjugates observed in control and smoke-exposed grapes at maturity (Table 1 and Table S1) were not preserved during fermentation (Table 1 and Table S2). The most abundant glycoconjugates, cresol, guaiacol and phenol pentose glucosides and syringol glucose glucosides, were detected in control grapes at 73, 38, 35 and 23 µg/kg, and in HS grapes at 988, 803, 576 and 535 µg/kg, respectively (Table S1). In contrast, guaiacol pentose glucoside and syringol glucose glucoside were present in control wines at 15 and 24 ug/L, while all other glycoconjugates were ≤6.3 µg/L (Table S2). Elevated levels of guaiacol pentose glucoside and syringol glucose glucoside (234 and 413 µg/L) remained in HS wines after fermentation, but the most abundant glycoconjugates of phenol and cresol were then rutinosides, at 59 and 102 µg/L, respectively (Table S2). The metabolic fates of cresol and phenol pentose glucosides are unclear, given the low cresol and cresol glucoside concentrations in HS wine do not support significant hydrolysis (unless cresols are further metabolized), and phenol was not measured in any of the wines.

To date, Caffrey and colleagues have published the only other study that measures changes in volatile phenol glycoconjugates during winemaking [17]. Of the 31 glycoconjugates measured (a mix of mono-, di- and trisaccharides), 20 decreased in concentration, five increased in concentration and six were not significantly different in concentration, from juice after pressing to wine after bottling; the largest changes in glycoconjugate concentrations were observed during the first half of primary fermentation, when glycosidase activities of *Saccharomyces* yeast were highest. However, it is difficult to make direct comparisons with the current study, given few glycoconjugates of guaiacol or syringol were detected, and the scope of the project was limited to analysis of glycoconjugates (i.e., no volatile phenol or sensory data were reported for finished wine) [17]. As smoke taint research has progressed over the past ~15 years, the suite of volatile phenols used as smoke taint markers has evolved, with new methods for direct and indirect measurement of free and bound volatile phenols being developed. Differences in both the volatile phenols measured and the methods used to measure them (in free and/or bound forms) complicate comparisons amongst the scientific literature on this topic. This is likely to continue in the short–medium term, as analytical methods for smoke taint are refined, particularly if new marker compounds and/or alternate storage forms of marker compounds are identified.

### 2.3. Influences of in-Canopy Misting and Smoke Density on the Degree of Smoke Taint in Grapes and Wine

As expected, few compositional differences were observed between control grapes with and without in-canopy misting (i.e., “CM” and “C” grapes), irrespective of sampling time (Table 2, Tables S1 and S3). While there were no significant differences in the volatile phenol concentrations of smoke-exposed grapes with and without misting (i.e., “HSM” and “HS” grapes) at maturity (i.e., at t = 4, Table 2), HSM grapes sampled 1 h after smoke exposure (i.e., at t = 1) comprised significantly lower volatile phenol levels than HS grapes (Table S3). Furthermore, at t = 4, the concentrations of several volatile phenol glycoconjugates were significantly lower in HSM grapes (than HS grapes), including pentose glucosides of guaiacol, 4-methylguaiacol, phenol and syringol (Table S1). These findings suggest in-canopy misting partially mitigated the uptake of volatile phenols by grapes during grapevine smoke exposure. 

Excluding 4-methylsyringol, which was not detected in any of the wines, the concentrations of volatile phenols were significantly higher in HS and HSM wines than in C and CM wines (Table 2). However, the only significant difference observed amongst the volatile phenol levels of HS and HSM wines was for 4-methylguaiacol, being 4 vs. 3 µg/L, respectively. With the exception of guaiacol glucose glucoside, the concentrations of volatile phenol glycoconjugates were again found to be significantly lower in HSM wines compared to HS wines (Table S2). This provides further evidence that in-canopy misting partially mitigated the uptake of volatile phenols during grapevine smoke exposure, but as discussed below, did not affect the sensory perception of smoke taint in HSM wines.

**Figure 2:**
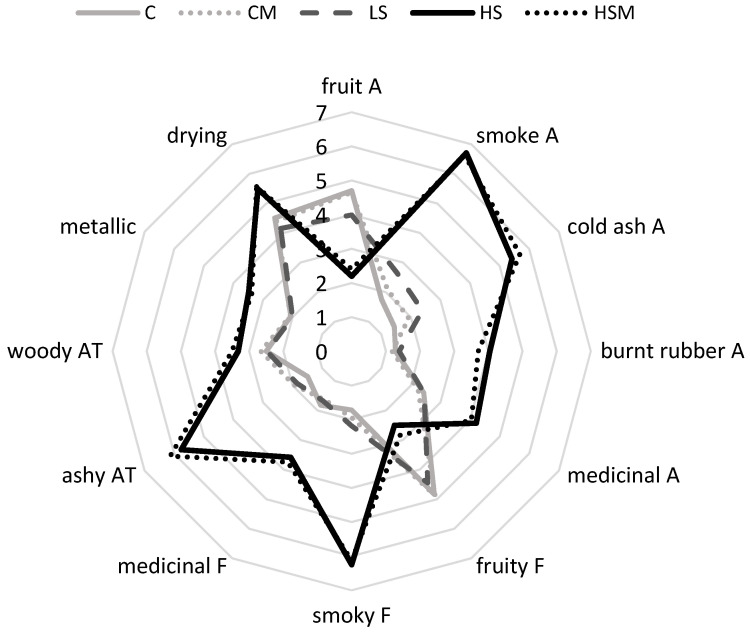
Sensory profiles of control and smoke-affected wines; A = aroma; F = flavor; AT = aftertaste. C = control (no smoke exposure); CM = control with misting; LS = low density smoke exposure; HS = high density smoke exposure; HSM = high density smoke exposure with misting. Values are mean intensity ratings of one wine per treatment, presented to 50 judges; ratings for each attribute were statistically significant (*P* = 0.05, one-way ANOVA).

The difference in smoke density achieved during LS and HS treatments significantly influenced the compositions of grapes and wines. LS grape juice contained far lower levels of volatile phenols than HS grape juice at t = 1, and in some cases at t = 2 and t = 3 also (Table 1), as well as lower volatile phenol glycoconjugate levels, especially at t = 4 (Table 1 and Table S1). However, as indicated above, only the phenol and cresol concentrations of LS grapes were significantly different from control grapes at t = 1 (Table 1). One-way ANOVA confirmed significant (albeit relatively small) differences in the glycoconjugate content of LS and C grapes (Table S1), specifically: guaiacol rutinoside (*P* = 0.015), 4-methylguaiacol rutinoside (*P* = 0.019), phenol pentose glucoside (*P* = 0.025), phenol rutinoside (*P* = 0.009) and cresol rutinoside (*P* = 0.004). HS wines contained significantly higher levels of volatile phenols and volatile phenol glycoconjugates than LS wines (Table 1 and Table S2), whereas the presence of low levels of cresols (1–3 µg/L) in LS wines differentiated them from control wines (Table 2). One-way ANOVA also confirmed the concentration of 13 of the 17 glycoconjugates reported in Table S2 were significantly higher in LS wines than in control wines, including: guaiacol pentose glucoside (*P* = 0.047), phenol rutinoside (*P* = 0.007), cresol rutinoside (*P* = 0.005), syringol glucose glucoside (*P* = 0.032) and syringol pentose glucoside (*P* = 0.017). Collectively, these results provide compositional evidence of low level smoke taint in LS wine, in agreement with sensory analysis results (Figure 2).

The sensory profiles of wines made from control (C and CM) and LS grapes were quite similar and comprised the most intense fruit aromas and flavors, and least intense smoke-related attributes (Figure 2). Few significant differences were perceived amongst these wines. The intensity of smoke aroma in the LS wine was slightly higher than in the control wine (Figure 2, Table S4), and provided the only sensory evidence of smoke taint, but was not significantly different from the CM wine and was considerably lower than for HS and HSM wines. The CM wine was also found to exhibit increased hotness (Table S4), due to the higher alcohol content of this wine (Table S5). In contrast to C, CM and LS wines, the sensory profiles of HS and HSM wines were dominated by smoke-related aromas and flavors, an ashy aftertaste and drying finish, which significantly diminished fruit intensity (Figure 2, Table S4); i.e., these wines were noticeably tainted by smoke, in agreement with wine volatile phenol data (Table 2). The only significant sensory difference observed between HS and HSM wines was the perception of hotness (Table S4), which was rated lower in HS wine compared to HSM wine, reflecting the lower alcohol content of HS wine (Table S5). As such, despite appearing to partially mitigate the uptake of smoke-derived volatile phenols by grapes, misting did not significantly influence the sensory perception of smoke taint in wine. Further research is needed to determine whether or not optimization of other factors, such as water droplet size and/or flow rate, might improve the efficacy of misting.

### 2.4. Concentration of Particulate Matter During Grapevine Exposure to Smoke

**Figure 3:**
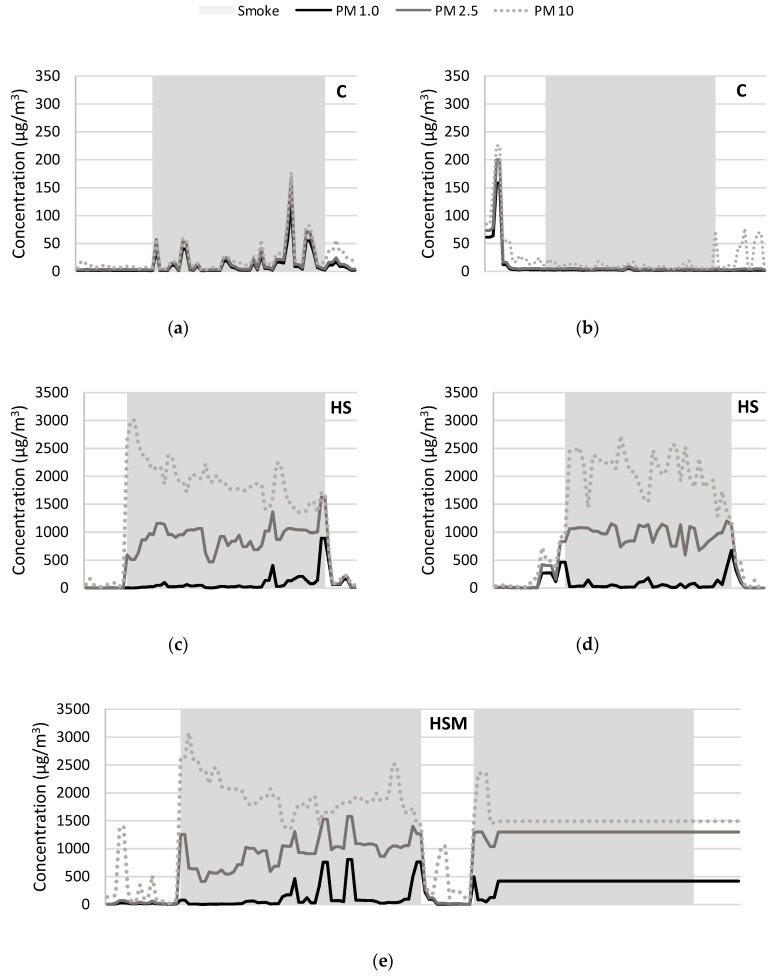

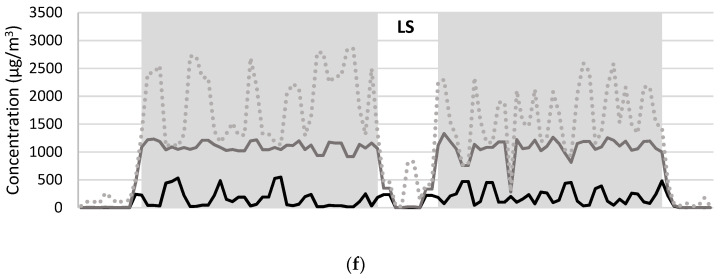
Particulate matter (PM_1.0_, PM_2.5_ and PM_10_) concentrations measured during field trials. The x axes reflect time, with shading indicating the 1 h window of each smoke treatment: (**a**–**d**) show PM data recorded during the high density smoke exposure (HS) treatments, with sensors positioned amongst the control vines and within the smoke tent, respectively (sensor positions were swapped between the two duplicate HS treatments); (**e**,**f**) show PM data recorded during the duplicate high smoke with misting (HSM) and low smoke (LS) treatments, respectively (with sensors again positioned within the smoke tent).

Particulate matter (PM) concentrations were measured during field trials to monitor smoke emission and exposure (Figure 3), using two commercial sensors: one positioned amongst control vines and one within the smoke tent. In the two days prior to field trials, PM_1.0_, PM_2.5_ and PM_10_ levels were ≤6.2, 13.6 and 89.7 µg/m^3^ respectively (data not shown). During field trials, PM levels detected by sensors positioned amongst control vines were typically <100 µg/m^3^ (e.g., Figure 3a,b), reflecting occasional, but minimal smoke drift. In contrast, elevated PM levels were detected by sensors positioned inside the smoke tent, for the duration of smoke treatments (Figure 3c–f). PM levels increased as soon as fuel was combusted to produce smoke, with PM_10_ > PM_2.5_ > PM_1.0_, in agreement with particle size distributions previously reported for smoke from domestic wood fires [29]. As expected, PM levels then decreased when smoke production stopped. The occasional PM signals (especially PM_10_) that were observed either before or after smoke treatments (e.g., as seen in Figure 3b,e,f) can be attributed to the movement of either smoke tents or sensors. During HS treatments (with or without misting), PM_2.5_ and PM_10_ levels approximated 1000 and 2000–2500 µg/m^3^, respectively (Figure 3c–e), but considerable signal variation was observed, which likely reflects a combination of the recurring combustion of fuel, detector saturation and the algorithm behind data acquisition. Detector saturation has been reported in previous smoke taint research [24], and in the current study, one of the sensors stopped acquiring PM data when its detector became fouled during the second HSM treatment (Figure 3e). Interestingly, the PM_2.5_ and PM_10_ levels detected during the LS treatment (Figure 3f) were similar to those from the HS and HSM treatments (Figure 3c–e); the increased fluctuation observed in the PM_10_ signal again reflects the recurring combustion of fuel (and smaller amounts of fuel compared with that combusted during HS and HSM treatments). These results suggest the levels of PM generated during LS treatments were still at (for PM_2.5_), or near (for PM_10_), the detector saturation levels, such that the sensors did not differentiate low and high smoke treatments as readily as was expected given the obvious visual differences in smoke density. The density of smoke applied to grapevines during HS and HSM treatments was likely far higher than would occur in vineyards during most bushfires, and where similar levels of smoke exposure do occur, the resulting taint should be easy to detect, either by chemical or sensory analysis. In contrast, the density of smoke applied to LS grapevines was readily detected by the environmental sensors, despite yielding wine in which the presence of smoke taint was difficult to detect by chemical or sensory analysis. The sensors could therefore be used to monitor the presence of smoke in vineyards during bushfires, and where vineyard exposure to smoke is detected, the need for grape compositional analysis to determine the presence of volatile phenols (and/or their glycoconjugates) as markers of smoke taint, based on both the duration and density of smoke exposure.

The environmental sensors also recorded temperature and relative humidity during the field trials (data not shown). Temperatures within the smoke tents increased by ~10 °C (relative to ambient temperature) during smoke treatments. Relative humidity differed more between the two days during which field trials were undertaken (i.e., from ~25–30% to ~40–55%) than between treatments, with the exception of the HSM treatment, during which the relative humidity in the smoke tent increased from 25–30% to 40%, due to the in-canopy misting. However, the differences observed in microclimate conditions were not expected to have significant or lasting effects on grapevine physiology, especially relative to the known effects of smoke on grapevine physiology [5]. 

## 3. Materials and Methods 

### 3.1. Chemicals

Chemicals (analytical grade) were purchased from Sigma Aldrich (Castle Hill, NSW, Australia). Solvents (HPLC grade) were sourced from Sigma Aldrich or Merck (Darmstadt, Germany). Deuterium-labelled internal standards (*d*_3_-guaiacol, *d*_4_-guaiacol, *d*_3_-4-methylguaiacol, *d*_7_-*o*-cresol, *d*_3_-syringol, *d*_3_-syringol gentiobioside) were synthesized in house, as previously reported [11,13,18,30].

### 3.2. Field Trials

Field trials involved the application of smoke (with or without in-canopy misting) to Cabernet Sauvignon grapevines (*Vitis vinifera*) growing in two adjacent rows of a vineyard located at the University of Adelaide’s Waite Campus in Urrbrae, South Australia (34°58′ S, 138°38′ E). Grapevines were planted (in 1998) in north-south aligned rows on their own roots (at 2.0 and 3.3 m vine and row spacings, respectively), trained to a bilateral cordon-vertical shoot positioned trellis system, hand-pruned to a two-node spur system and drip-irrigated twice weekly from fruit set to pre-harvest. Treatments comprised: (i) a control (C), i.e., no smoke exposure; (ii) a low smoke treatment (LS), i.e., exposure to low density smoke; (iii) a high smoke treatment (HS), i.e., exposure to high density smoke; (iv) a control with misting (CM), i.e., in-canopy misting but no smoke exposure; and (v) a high smoke treatment with misting (HSM), i.e., exposure to high density smoke with in-canopy misting. 

Smoke treatments involved grapevines being exposed to smoke for 1 h (at approximately 7 d post-veraison), using purpose-built smoke tents (6.0 × 2.5 × 2.0 m) and experimental conditions similar to those described previously [3,4,5]; except that barley straw was combusted in two commercial fire box smokers, positioned at each end of the smoke tent. Low and high density smoke treatments were achieved by burning approximately 1.5 and 5 kg of barley straw respectively, with fuel added at regular intervals (i.e., every ~10 min) to ensure smoke production throughout the duration of treatment. Control and smoke-exposed vines were separated by at least one buffer vine. In-canopy misting treatments involved the continuous application of fine (65 µm) water droplets to the bunch zone of six adjacent vines, using a purpose-built sprinkler system (comprising two CoolNet Pro “tee” configuration sprinklers (Netafim Australia, Adelaide, SA, Australia) per vine, suspended 30 cm above the cordon, each delivering water at a rate of 11 L/h) supplied with mains water pumped from a 1000 L plastic tank, as described previously [22]. 

**Figure 4:**
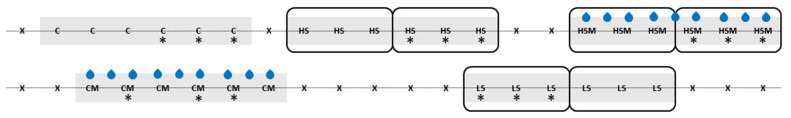
Schematic diagram of treatments (C = control (no smoke exposure); CM = control with misting; LS = low density smoke exposure; HS = high density smoke exposure; HSM = high density smoke exposure with misting), showing the positioning of smoke tents, in-canopy sprinklers (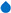), vine replicates (*) and buffer vines (×), within the two adjacent rows of Cabernet Sauvignon vines.

Whereas smoke treatments were applied to panels of vines in triplicate in previous trials [5,6,16,27], fire bans imposed by the state government (due to increased fire danger ratings associated with hot, dry and/or windy weather conditions) limited the number of smoke treatments that could be applied in the current study. Field trials were also constrained by where the in-canopy sprinkler systems were installed. As such, each treatment was applied to six adjacent grapevines, as depicted in Figure 4. LS, HS and HSM treatments comprised duplicate applications of smoke to three adjacent vines at a time, with the in-canopy sprinkler system turned on 5 min before the first HSM treatment was applied, and off 15 min after the second HSM treatment was completed, such that CM and HSM grapevines were misted for approximately 2.5 h in total. Three vine replicates per treatment were subsequently selected for berry sampling and winemaking (with vine replicates becoming wine replicates, for each treatment). With the exception of CM, three adjacent vines were chosen as vine replicates; for LS, HS and HSM, vine replicates were from the same smoke application. In the case of CM, the selection of non-adjacent vine replicates accounted for missing sprinklers. 

Two portable environmental sensors (R9 series, Attentis Pty. Ltd., Cheltenham, Vic., Australia) were used to monitor temperature, relative humidity, and the concentration of particulate matter (PM_1.0_, PM_2.5_ and PM_10_) during field trials. One sensor was positioned inside the smoke tent during each smoke treatment, while the other sensor was positioned mid-row, amongst control (C and CM) vines. Environmental data were captured continuously (typically at 1–2 min intervals) and uploaded to the manufacturer’s network via an internal Wi-Fi connection. Data were subsequently exported from the network as Excel files.

Samples (50 berries, from each of the three vine replicates per treatment, chosen randomly according to a previously published sampling protocol [31]) were collected at five time points: (i) immediately prior to smoke exposure (t = 0); (ii) 1 h after smoke exposure (t = 1); (iii) 1 day after smoke exposure, (t = 2); (iv) 7 days after smoke exposure, (t = 3); and (v) 4 weeks after smoke exposure (t = 4) at maturity. Samples were homogenized (T18 Ultra Turrax, IKA, Staufen, Germany) and frozen at –4 °C until quantitation of volatile phenols and volatile phenol glycoconjugates (approximately 1 month after sampling). The remaining control and smoke-exposed fruit were harvested (4 weeks after smoke exposure) for winemaking, with the vine replicates from each treatment becoming wine replicates. Grapes were intended to be harvested when TSS levels were approximately 24 °Brix, with maturity sampling performed on samples (50 berries) collected from buffer vines. However, analysis of juice following harvest and crushing of grapes indicated significant variation in maturity amongst vine replicates, with average TSS levels ranging from 19.6 to 22.3 °Brix (Table S6). Viticultural data were collected to evaluate variation in vine physiology and while significant differences were not observed for TSS, bunch number, yield, shoot number or pruning weight between treatments (Table S6), this was attributed to the large relative standard errors (i.e., 10–25.9%) associated with one or more treatments, for each measurement. Vine variation likely explains the different TSS levels observed amongst juice samples, and therefore the differences in wine alcohol content, which were perceived by the sensory panel (Figure 2, Table S4). Nevertheless, the differences in the intensity of hotness between wines were not considered to have significantly affected the panel’s perception of smoke taint. Other significant differences in basic wine composition (i.e., titratable acidity (TA) and color; Table S5) did not significantly affect the panel’s rating of acidity (Table S4) or were addressed by presenting wines to panelists monadically. 

### 3.3. Preparation of Acid Hydrolysates

Juices from HS grape homogenate samples (two replicates from each time point) were subjected to strong acid hydrolysis, using methodology similar to that reported by Noestheden and colleagues [15]. Briefly, aliquots of homogenate (10 g) were centrifuged for 30 min at 3500× *g* (Universal 320R centrifuge, Andreas Hettich GmBH and Co. KG, Tuttlingen, Germany), and 2 mL of the resulting juice was purified by solid phase extraction (using Strata X 33 µm polymeric reversed phase cartridges, 200 mg/3 mL; Phenomenex, Lane Cove, NSW, Australia). Samples were eluted with 40% acetonitrile in water (2 mL), dried (under nitrogen at 35 °C), reconstituted in 2 mL water and acidified to pH ~1 (via dropwise addition of 1 M hydrochloric acid), before being heated at 100 °C for 4 h in 8 mL PTFE tubes (SPI Supplies, West Chester, PA, USA). Hydrolysates were subsequently cooled to ambient temperature, pH adjusted back to wine pH (i.e., pH 3.0–3.5, via dropwise addition of 1 M aqueous sodium hydroxide) and frozen prior to chemical analysis. 

### 3.4. Winemaking

Bunches (5 kg per replicate, per treatment, chosen randomly) were crushed and de-stemmed, with the addition of 50 mg/L sulfur dioxide (added as an 8% solution of potassium metabisulphite). Tartaric acid was added to adjust the pH of must to 3.5, prior to inoculation with 150 mg/L of PDM yeast (Maurivin, AB Biotek, Sydney, NSW, Australia) and the addition of diammonium phosphate (100 mg/L). Musts were fermented on skins at ambient temperature (25–27 °C), with the cap plunged twice daily. When wines approached dryness (2 g/L residual sugar), they were pressed and held at 25 °C until completion of fermentation (i.e., until residual sugars approached 0 g/L), after which they were racked from gross lees and cold stabilized (at 0 °C for 4 weeks). No wines underwent malolactic fermentation. Wine pH and free SO_2_ were adjusted to 3.5 and 30 mg/L respectively, before bottling (in 375 mL glass bottles, with screw cap closures). Bottles were stored at 15 °C for two months prior to sensory analysis. Prior to bottling, wines were sampled for chemical analysis.

### 3.5. Chemical Analysis of Grapes, Wine and Acid Hydrolysates

Residual sugars were measured enzymatically (using a glucose/fructose enzymatic test kit from Vintessential Laboratories Pty. Ltd., Dromana, Vic., Australia) using a Chemwell 2910 automated analyzer (Awareness Technology Inc., Palm City, FL, USA). pH and titratable acidity (TA, expressed as g/L tartaric acid) were measured using a Mettler Toledo T50 autotitrator coupled to a Mettler Toledo InMotion Flex autosampler (Port Melbourne, Vic., Australia). Ethanol content (% alcohol by volume, abv) was measured with an alcolyzer (Anton Paar, Graz, Austria). Wine color density, wine hue and total phenolics were determined by the modified Somers color assay [32] using an Infinite^®^ 200 PRO spectrophotometer (Tecan, Männedorf, Switzerland). 

#### 3.5.1. Determination of Volatile Phenols

The concentrations of volatile phenols (guaiacol, 4-methylguaiacol, phenol, *o-*, *m-* and *p*-cresol, syringol and 4-methylsyringol) were measured in grape juice and wine samples, using stable isotope dilution analysis (SIDA) methods described previously [13,18,30], with the method developed for analysis of wine also used for acid hydrolysates. These publications describe the preparation of isotopically labelled standards (*d*_4_-guaiacol and *d*_3_-syringol for analysis of grape juice performed at the University of Adelaide and *d*_3_-guaiacol, *d*_3_-4-methylguaiacol, *d*_7_-*o*-cresol and *d*_3_-syringol for analysis of wine and acid hydrolysates, performed by the Australian Wine Research Institute’s (AWRI) Commercial Services Laboratory, Adelaide, Australia), and method validation and instrumental operating conditions. All measurements were performed using an Agilent 6890 gas chromatograph coupled to a 5973 mass spectrometer (Agilent Technologies, Forest Hill, Vic., Australia). The limit of quantitation for volatile phenols was 1–2 µg/L.

#### 3.5.2. Determination of Volatile Phenol Glycosides

The concentrations of volatile phenol glycosides were measured in grape (homogenate), wine and acid hydrolysate samples, as syringol glucose glucoside (gentiobioside) equivalents, using liquid chromatography-tandem mass spectrometry (HPLC–MS/MS) according to previously published SIDA methods [11,13]; the method developed for analysis of wine was also used for acid hydrolysates. Glycoconjugate analyses were performed on an Agilent 1200 high performance liquid chromatograph (HPLC) equipped with a 1290 binary pump, coupled to an AB SCIEX Triple Quad^TM^ 4500 tandem mass spectrometer, with a Turbo V^TM^ ion source (Framingham, MA, USA). Data acquisition and processing were performed using Analyst software (version 1.7 AB SCIEX). The preparation of the isotopically labelled internal standard (*d_3_*-syringol gentiobioside), method validation and instrumental operating conditions were as previously reported [11,13]. The limit of quantitation for volatile phenol glycosides was 1 µg/kg (as syringol glucose glucoside equivalents). 

### 3.6. Sensory Analysis of Wine

The replicate wines from each treatment were assessed by a group of sensory experts from the University of Adelaide (for evidence of faults or obvious differences between replicates), before replicates were blended. The sensory profiles of wines (as one wine per treatment) were then determined using the rate-all-that-apply (RATA) method [33] and a panel comprising staff and students from the University of Adelaide and AWRI, and regular wine consumers (*n* = 50, 12 male and 38 female, aged between 20 and 74 years). Prior to wine evaluation, panelists completed a brief induction, during which they were familiarized with both the RATA procedure and a list of attributes and their definitions (Table S7), which were adapted from previous studies [5,26]. RATA assessments were conducted in sensory booths at 22–23 °C under sodium lights, with wine aliquots (30 mL) presented monadically, in a randomized order, in covered, 3-digit coded 215 mL stemmed International Organization for Standardization wine glasses. Panelists rated the intensity of each sensory attribute using line scales (where 0 = “not perceived”, 1 = “extremely low” and 9 = “extremely high”). Panelists rinsed thoroughly with pectin solution (1 g/L) and rested for at least 1 min between samples, with water and plain crackers provided as palate cleansers. Data were acquired with Red Jade software (Redwood Shores, CA, USA).

### 3.7. Data Analysis

Chemical data were analyzed by one and two-way analysis of variance (ANOVA) using GenStat (19th Edition, VSN International Limited, Herts, UK). Sensory data were analyzed using SenPAQ (version 5.01, Qi Statistics, Reading, UK) and XLSTAT (version 2018.1.1, Addinsoft, New York, NY, USA). Mean comparisons were performed by Fisher’s least significant difference (LSD) multiple comparison test at *P* < 0.05. 

## Figures and Tables

**Table 1:** Concentrations of volatile phenols in juice (µg/L) and volatile phenol glycosides in homogenate (µg/kg) from control and smoke-exposed grapes sampled from pre-smoke exposure (t = 0) to maturity (t = 4), and in corresponding wines (µg/L); different densities of smoke were achieved by burning different amounts of fuel.

Treatment/ Timepoint		Guaiacol	4-MethylGuaiacol	Phenol	Cresols	Syringol	4-MethylSyringol	Guaiacol Glycosides	4-MethylGuaiacol Glycosides	Phenol Glycosides	Cresol Glycosides	Syringol Glycosides	4-MethylSyringol Glycosides
C	t = 0	1.9 b	3.6	1.5	2.6	12 b	2.5	3.9 b	1.5 b	3.1 b	12 b	4.1 b	nd
	t = 1	9.5 a	4.1	2.6	5.1	21 a	3.0	5.5 b	2.1 b	3.8 b	16 b	5.9 b	1.1 b
	t = 2	2.4 b	3.6	1.6	2.7	8.4 b	2.0	8.4 b	3.0 b	4.7 b	26 b	14 b	2.2 b
	t = 3	1.9 b	3.6	1.6	2.4	7.9 b	1.8	13 b	4.6 b	8.0 b	31 b	30 ab	3.6 b
	t = 4	2.2 b	3.6	1.6	2.4	13 b	1.8	44 a	22 a	45 a	83 a	44 a	13 a
	P	0.033	ns	ns	ns	0.017	ns	0.002	<0.001	<0.001	<0.001	0.037	0.011
LS	t = 0	1.7 b	3.5 b	1.4 c	2.5 c	6.2 c	2.0 b	3.5 b	1.1 b	3.6 b	9.0 c	3.1 d	nd
	t = 1	12 a	4.1 a	6.9 a	12 a	25 a	2.9 a	6.4 b	2.1 b	5.3 b	20 bc	12 cd	1.5 b
	t = 2	2.8 b	3.6 b	4.7 b	4.9 b	6.0 c	1.9 b	14 b	4.8 b	16 b	46 b	27 bc	3.6 b
	t = 3	2.6 b	3.6 b	5.1 ab	4.8 b	13 b	1.8 b	16 b	6.3 b	26 b	47 b	42 b	4.4 b
	t = 4	3.1 b	3.6 b	6.3 ab	5.0 b	11 bc	1.8 b	73 a	38 a	121 a	154 a	77 a	18 a
	P	<0.001	<0.001	0.001	<0.001	<0.001	<0.001	<0.001	<0.001	<0.001	<0.001	<0.001	<0.001
HS	t = 0	1.8 c	3.5 b	1.8 b	2.7 b	7.8 b	1.9 b	3.2 b	1.4 b	3.3 b	10 b	3.5 c	nd
	t = 1	108 a	20 a	55 a	83 a	126 a	17 a	45 b	14 b	22 b	98 b	71 c	11 b
	t = 2	25 b	5.1 b	12 b	23 b	24 b	2.7 b	158 b	51 b	69 b	263 b	310 bc	48 b
	t = 3	12 c	4.6 b	17 b	18 b	12 b	1.9 b	229 b	70 b	144 b	316 b	526 ab	69 b
	t = 4	10 c	4.2 b	21 b	13 b	12 b	1.8 b	894 a	297 a	745 a	1118 a	843 a	248 a
	P	<0.001	<0.001	0.012	<0.001	<0.001	<0.001	0.001	<0.001	<0.001	<0.001	0.001	<0.001
P ^1^		<0.001	<0.001	<0.001	<0.001	<0.001	<0.001	<0.001	<0.001	<0.001	<0.001	<0.001	<0.001
LSD ^1^		15.6	3.7	14.2	15.0	24.6	3.3	182.0	52.6	112.6	61.1	172.8	36.0
C	wine	1.7 b	nd	–	nd	1.7 b	nd	19 b	4.2 b	6.2 b	7.5 b	30 b	1.4 b
LS	wine	4.3 b	nd	–	5.9 b	2.7 b	nd	30 b	7.7 b	17 b	15 b	53 b	2.3 b
HS	wine	29 a	4.0	–	28 a	4.7 a	nd	283 a	68 a	112 a	115 a	501 a	30 a
P		<0.001	–	–	<0.001	0.011	–	0.002	0.001	<0.001	<0.001	0.002	<0.001

C = control (no smoke exposure); LS = low density smoke exposure; HS = high density smoke exposure. Values are means of three replicates (*n* = 3); nd = not detected. Different letters (within columns) indicate statistical significance (*P* = 0.05, one way ANOVA) amongst: (i) time points (i.e., immediately prior to smoke exposure (t = 0); 1 h after smoke exposure (t = 1); 1 day after smoke exposure (t = 2); 7 days after smoke exposure (t = 3); and 4 weeks after smoke exposure (t = 4) being maturity) for grape data; and (ii) wines; ns = not significant. ^1^ *P* and LSD values for two-way ANOVA of grape data, by treatment and time. Phenol was not measured in wines.

**Table 2:** Concentrations of volatile phenols in juice (µg/L) from control and smoke-exposed grapes at maturity (t = 4), and in corresponding wines (µg/L), with and without in-canopy misting; different densities of smoke were achieved by burning different amounts of fuel.

Volatile Phenols		C	CM	LS	HS	HSM	P
juice	guaiacol	2.2 ± 0.1 b	2.4 ± 0.1 b	3.1 ± 0.1 b	10 ± 1.2 a	7.6 ± 1.9 a	<0.001
	4-methylguaiacol	3.6 ± 0.1 b	3.5 ± 0.1 b	3.6 ± 0.1 b	4.2 ± 0.1 a	4.0 ± 0.2 a	0.003
	phenol	1.6 ± 0.3 b	1.9 ± 0.2 b	6.3 ± 0.9 b	21 ± 4.1 a	17 ± 2.9 a	<0.001
	cresols	2.4 ± 0.1 b	2.7 ± 0.1 b	5.0 ± 0.7 b	13 ± 2.1 a	12 ± 1.5 a	<0.001
	syringol	13 ± 0.6	12 ± 1.1	11 ± 0.7	12 ± 0.9	13 ± 0.7	ns
	4-methylsyringol	1.8 ± 0.1	1.8 ± 0.1	1.8 ± 0.1	1.8 ± 0.1	1.9 ± 0.1	ns
wine	guaiacol	1.7 ± 1.0 b	1.0 ± 0.7 b	4.3 ± 0.1 b	29 ± 0.3 a	23 ± 4.9 a	<0.001
	4-methylguaiacol	nd	nd	nd	4.0 ± 0.1 a	3.0 ± 0.6 b	<0.001
	*o*-cresol	nd	nd	2.7 ± 0.1 b	11 ± 0.3 a	11 ± 1.7 a	<0.001
	*m*-cresol	nd	nd	1.9 ± 0.1 b	10 ± 0.1 a	10 ± 1.9 a	<0.001
	*p*-cresol	nd	nd	1.3 ± 0.1 b	6.7 ± 0.3 a	5.0 ± 1.2 a	<0.001
	syringol	1.7 ± 1.0 b	2.0 ± 0.1 b	2.7 ± 0.1 b	4.7 ± 0.3 a	4.7 ± 0.7 a	<0.001
	4-methylsyringol	nd	nd	nd	nd	nd	*–*

C = control (no smoke exposure); CM = control with misting; LS = low density smoke exposure; HS = high density smoke exposure; HSM = high density smoke exposure with misting. Values are means of three replicates (*n* = 3) ± standard errors. Different letters (within rows) indicate statistical significance (*P* = 0.05, one-way ANOVA); ns = not significant.

## Supplementary Materials

The following are available online: Table S1: Concentrations (µg/kg) of volatile phenol glycoconjugates in control and smoke-exposed grapes sampled from pre-smoke exposure (t = 0) to maturity (t = 4). Table S2: Concentrations (µg/L) of volatile phenol glycoconjugates in wines made from control and smoke-exposed grapes. Table S3: Concentrations (µg/L) of volatile phenols in juice from control and smoke-exposed grapes sampled from pre-smoke exposure (t = 0) to maturity (t = 4). Table S4: Mean intensity ratings for sensory attributes of control and smoke-affected wines. Table S5: Basic composition of control and smoke-affected wines. Table S6: Viticultural measurements for control and smoke-affected grapevines. Table S7: Aroma and palate attributes used in sensory analysis of wines.
