# Supplementary material for: Uptake and Glycosylation of Smoke-Derived Volatile Phenols by Cabernet Sauvignon Grapes and Their Subsequent Fate during Winemaking
Source: Molecules. 2020 Aug 14;25(16):3720. doi: 10.3390/molecules25163720 (PMC7464031; doi:10.3390/molecules25163720)
Supplement: Supplementary file 1 [file molecules-25-03720-s001.pdf]

Supplementary Materials

Uptake and Glycosylation of Smoke-Derived Volatile Phenols by Cabernet Sauvignon Grapes and their Subsequent Fate during Winemaking

Colleen Szeto [1,2], Renata Ristic [1,2], Dimitra Capone [1,2], Carolyn Puglisi [1], Vinay Pagay [1,2], Julie Culbert [3], WenWen Jiang [3], Markus Herderich [1,2,3], Jonathan Tuke [4] and Kerry Wilkinson [1,2,*]

[1] The University of Adelaide, School of Agriculture, Food and Wine, PMB 1, Glen Osmond, SA, 5064, Australia; colleen.szeto@adelaide.edu.au (C.S.); renata.ristic@adelaide.edu.au (R.R.); dimitra.capone@adelaide.edu.au (D.C.); carolyn.puglisi@adelaide.edu.au (C.P.); vinay.pagay@adelaide.edu.au (V.P.)
[2] The Australian Research Council Training Centre for Innovative Wine Production, PMB 1, Glen Osmond, SA, 5064, Australia
[3] The Australian Wine Research Institute, PO Box 197, Glen Osmond, SA, 5064, Australia julie.culbert@awri.com.au (J.C.); maddy.jiang@awri.com.au (W.J.); markus.herderich@awri.com.au (M.H.);
[4] School of Mathematical Sciences, The University of Adelaide, SA 5000, Australia; simon.tuke@adelaide.edu.au
* Correspondence: kerry.wilkinson@adelaide.edu.au; Tel.: +61-8-8313-7360

Table S1. Concentrations (μg/kg) of volatile phenol glycoconjugates in control and smoke-exposed grapes sampled from pre-smoke exposure (t = 0) to maturity (t = 4).

Treatment/	Timepoint	GuG	GuGG	GuPG	GuR	4MGuG	4MGuPG	4MGuR	PhG	PhGG	PhPG	PhR	CrG	CrGG	CrPG	CrR	SyG	SyGG	SyPG	MSyGG	MSyPG
C	t = 0	nd	nd	3.7 b	nd	nd	tr	tr	nd	nd	2.5 b	tr	nd	tr	9.2 b	2.0	tr	2.7	tr	tr	nd
	t = 1	tr	nd	5.0 b	nd	nd	1.2 b	tr	tr	nd	3.0 b	tr	1.1	tr	12 b	2.7	1.1 b	3.7	1.1 b	1.0 b	nd
	t = 2	tr	tr	7.3 b	nd	nd	1.7 b	1.3 b	nd	nd	3.7 b	tr	1.0	tr	19 b	4.9	1.6 b	11	2.0 b	2.1 b	nd
	t = 3	tr	tr	11 b	tr	nd	2.3 b	2.2 b	nd	tr	6.3 b	1.3 b	tr	tr	23 b	7.0	1.5 b	24	3.9 b	3.3 b	tr
	t = 4	1.8	1.9	38 a	2.2 a	1.8	10 a	10 a	1.6	1.0	35 a	7.3 a	nd	3.0	73 a	6.3	4.8 a	23	16 a	10 a	2.8
	P	–	–	<0.003	–	–	<0.001	<0.001	–	–	<0.001	<0.001	ns	–	<0.001	ns	0.003	ns	0.003	0.026	–
CM	t = 0	nd	nd	3.1 b	nd	nd	tr	1.0 b	tr	nd	2.6 b	tr	nd	tr	8.7 d	2.1	tr	2.3	tr	tr	nd
	t = 1	nd	nd	2.7 b	nd	nd	tr	tr	nd	nd	1.6 b	tr	nd	tr	8.4 d	1.8	tr	2.5	tr	tr	nd
	t = 2	nd	nd	3.7 b	nd	nd	tr	tr	nd	nd	3.5 b	tr	nd	tr	12 c	2.3	tr	3.6	1.2 b	tr	nd
	t = 3	nd	nd	3.7 b	tr	nd	tr	tr	nd	nd	3.9 b	tr	nd	tr	14 b	2.4	tr	4.4	1.3 b	tr	nd
	t = 4	1.9	0.7	17 a	1.2	1.6	6.9	7.8 a	1.4	nd	20 a	4.4	nd	2.9	43 a	3.3	2.7	6.7	6.8 a	2.7	1.7
	P	–	–	<0.001	–	–	–	<0.001	–	–	<0.001	–	–	–	<0.001	ns	–	ns	<0.001	–	–
LS	t = 0	nd	nd	3.4 b	nd	nd	tr	tr	tr	nd	2.8 b	tr	nd	tr	6.7 b	1.7 d	tr	2.3 c	tr	tr	nd
	t = 1	1.5 c	nd	4.0 b	nd	nd	1.0 b	1.0 b	tr	nd	4.0 b	1.0 b	3.0 b	nd	13 b	3.2 d	1.3 c	9.0 c	1.4 b	1.5 b	nd
	t = 2	3.0 a	tr	9.2 b	nd	nd	2.5 b	2.3 b	0.6 b	tr	13 b	2.3 b	10 a	tr	27 b	9.2 c	3.2 b	21 b	3.4 b	3.2 b	tr
	t = 3	1.2 c	tr	13 b	1.5 b	nd	3.0 b	3.3 b	0.5 b	tr	21 b	4.3 b	0.6 c	tr	33 b	12.6 b	1.4 c	35 a	5.3 b	4.0 b	tr
	t = 4	2.3 b	3.6	63 a	4.3 a	2.3	16 a	20 a	3.0 a	1.3	94 a	23 a	nd	2.2	136 a	15.0 a	6.5 a	43 a	27 a	14.5 a	3.7
	P	<0.001	–	<0.001	<0.001	–	<0.001	<0.001	ns	–	<0.001	<0.001	<0.001	–	<0.001	<0.001	<0.001	<0.001	<0.001	<0.001	–
HS	t = 0	nd	nd	3.1 b	nd	nd	tr	tr	tr	nd	2.6 b	tr	nd	tr	7.8 b	1.8 c	tr	2.3 c	tr	tr	nd
	t = 1	24	1.1 b	19 b	1.0 c	nd	9.2 b	4.5 b	1.0 b	tr	18 b	3.0 b	51 ab	tr	36 b	11 c	23 b	44 c	3.6 b	11 b	nd
	t = 2	35	5.7 b	115 b	2.7 c	nd	36 b	15 b	2.6 b	2.4 b	55 b	9.8 b	70 a	1.0 b	137 b	56 b	43 ab	248 b	19 b	44 b	4.2 b
	t = 3	23	10 b	185 b	11 b	nd	46 b	25 b	2.2 b	5.4 b	115 b	21 b	9.4 c	1.2 b	217 b	89 ab	22 b	455 a	49 b	62 b	7.1 b
	t = 4	20	45 a	803 a	25 a	8.4	171 a	118 a	14 a	20 a	576 a	135 a	27 bc	5.5 a	988 a	98 a	50 a	535 a	258 a	220 a	27 a
	P	ns	<0.001	<0.001	<0.001	–	0.002	<0.001	0.006	<0.001	<0.001	<0.001	0.001	<0.001	<0.001	<0.001	0.005	0.002	<0.001	<0.001	<0.001
HSM	t = 0	nd	nd	3.4 c	nd	nd	tr	tr	nd	nd	2.5 d	tr	nd	tr	9.6 c	2.3 c	tr	2.3 d	tr	tr	nd
	t = 1	9.0 b	tr	7.5 c	tr	nd	4.5 c	2.3 d	1.6 c	tr	8.7 cd	1.3 b	27 b	tr	22 c	5.9 c	6.6 c	18 d	2.0 d	4.0 c	tr
	t = 2	15 a	3.7 b	55 bc	1.9 c	nd	18 b	9.6 c	1.2 c	1.3 b	38 c	6.8 b	43 a	tr	96 bc	42 b	19 b	129 c	11 c	21 bc	2.4 b
	t = 3	15 a	6.2 b	87 b	8.1 b	nd	22 b	16 b	4.3 b	2.7 b	86 b	17 b	8.5 bc	tr	147 b	71 a	11 c	238 b	26 b	30 b	3.8 b
	t = 4	9.0 b	30 a	421 a	17 a	5.4	83 a	66 a	8.8 a	11 a	351 a	97 a	17 bc	4.2	673 a	69 a	32 a	325 a	136 a	115 a	16 a
	P	<0.001	<0.001	<0.001	<0.001	–	<0.001	<0.001	<0.001	<0.001	<0.001	<0.001	<0.001	–	<0.001	<0.001	<0.001	<0.001	<0.001	<0.001	<0.001
P[1]		<0.001	<0.001	<0.001	<0.001	<0.001	<0.001	<0.001	0.007	<0.001	<0.001	<0.001	<0.001	<0.001	<0.001	<0.001	<0.001	<0.001	<0.001	<0.001	<0.001
LSD[1]		7.2	16	307	5.0	2.4	68	27	6.5	6.6	173	34	6.6	1.2	303	20	14	172	80	62	5.9

C = control (no smoke exposure); CM = control with misting; LS = low density smoke exposure; HS = high density smoke exposure; HSM = high density smoke exposure with misting. Values are means of three replicates (n = 3) measured as syringol glucose-glucoside equivalents; nd = not detected; tr = trace (i.e. 0.5–1 μg/kg). Different letters (within columns, by treatment) indicate statistical significance (P = 0.05, one way ANOVA) amongst time points, i.e.: immediately prior to smoke exposure (t = 0); 1 hour after smoke exposure (t = 1); 1 day after smoke exposure (t = 2); 7 days after smoke exposure (t = 3); and 4 weeks after smoke exposure (t = 4) being maturity; ns = not significant. Gu = guaiacol; Cr = cresol; Ph = phenol; Sy = syringol; 4MG = 4-methylguaiacol; MSy = 4-methylsyringol; G = glucoside; GG = glucose-glucoside; PG = pentose-glucoside; R = rutinoside. [1] *P* and LSD values for one way ANOVA of data by treatment at maturity (t = 4).

Table S2. Concentrations (μg/L) of volatile phenol glycoconjugates in wines made from control and smoke-exposed grapes.

Treatment	GuG	GuGG	GuPG	GuR	4MGuPG	4MGuR	PhG	PhGG	PhPG	PhR	CrPG	CrR	SyG	SyGG	SyPG	MSyGG	MSyPG
C	tr	tr	15 c	3.3 c	2.3 c	2.0 c	1.8 c	tr	1.1 c	3.1 c	1.1 c	6.3 c	tr	24 c	4.8 c	tr	tr
CM	nd	tr	6.1 c	1.2 c	1.5 c	tr	1.0 c	nd	tr	1.7 c	tr	3.2 c	tr	11 c	1.8 c	tr	tr
LS	1.0 c	tr	21 c	6.6 c	3.3 c	4.4 c	3.9 c	tr	2.6 c	9.8 c	1.5 c	14 c	1.4 c	43 c	8.8 c	1.5 c	tr
HS	9.4 a	2.1	234 a	37 a	37 a	31 a	31 a	4.7 a	17 a	59 a	14 a	102 a	11 a	413 a	77 a	23 a	6.6 a
HSM	6.2 b	1.6	126 b	28 b	19 b	21 b	21 b	3.0 b	10 b	43 b	10 b	78 b	7.4 b	272 b	47 b	12 b	4.1 b
P	<0.001	ns	<0.001	<0.001	<0.001	<0.001	<0.001	<0.001	<0.001	<0.001	<0.001	<0.001	<0.001	<0.001	<0.001	<0.001	<0.001

C = control (no smoke exposure); CM = control with misting; LS = low density smoke exposure ; HS = high density smoke exposure; HSM = high density smoke exposure with misting. Values are means of three replicates (n = 3) measured as syringol glucose-glucoside equivalents; nd = not detected; tr = trace (i.e. 0.5–1 μg/L). Different letters (within columns) indicate statistical significance (P = 0.05, one way ANOVA); ns = not significant. Gu = guaiacol; Cr = cresol; Ph = phenol; Sy = syringol; 4MG = 4-methylguaiacol; MSy = 4-methylsyringol; G = glucoside; GG = glucose-glucoside; PG = pentose-glucoside; R = rutinoside. 4-Methylguaicol glucoside (4MGuG), cresol glucoside (CrG) and cresol glucose glucoside (CrGG) were not detected in any wine.

Table S3. Concentrations (µg/L) of volatile phenols in juice from control and smoke-exposed grapes sampled from pre-smoke exposure (t = 0) to maturity (t = 4).

Treatment/ Timepoint		Guaiacol	4-Methyl Guaiacol	Phenol	Cresols	Syringol	4-Methyl Syringol
C	t = 0	1.9 b	3.6	1.5	2.6	12 b	2.5
	t = 1	9.5 a	4.1	2.6	5.1	21 a	3.0
	t = 2	2.4 b	3.6	1.6	2.7	8.4 b	2.0
	t = 3	1.9 b	3.6	1.6	2.4	7.9 b	1.8
	t = 4	2.2 b	3.6	1.6	2.4	13 b	1.8
	P	0.033	ns	ns	ns	0.017	ns
CM	t = 0	1.7 b	3.5	1.5 b	3.2	8.6	2.0
	t = 1	2.6 a	3.5	1.5 b	2.8	8.0	1.9
	t = 2	1.9 b	3.5	3.4 a	2.8	21	1.9
	t = 3	1.9 b	3.5	1.8 b	2.8	8.8	1.8
	t = 4	2.4 a	3.5	1.9 b	2.7	12	1.8
	P	<0.001	ns	0.006	ns	ns	ns
LS	t = 0	1.7 b	3.5 b	1.4 b	2.5 c	6.2 c	2.0 b
	t = 1	12 a	4.1 a	6.9 a	12 a	25 a	2.9 a
	t = 2	2.8 b	3.6 b	4.7 a	4.9 b	6.0 c	1.9 b
	t = 3	2.6 b	3.6 b	5.1 a	4.8 b	13 b	1.8 b
	t = 4	3.1 b	3.6 b	6.3 a	5.0 b	11 bc	1.8 b
	P	<0.001	<0.001	0.001	<0.001	<0.001	<0.001
HS	t = 0	1.8 c	3.5 b	1.8 c	2.7 c	7.8 b	1.9 b
	t = 1	108 a	20 a	55 a	83 a	126 a	17 a
	t = 2	25 b	5.1 b	12 b	23 b	24 b	2.7 b
	t = 3	12 c	4.6 b	17 b	18 b	12 b	1.9 b
	t = 4	10 c	4.2 b	21 b	13 b	12 b	1.8 b
	P	<0.001	<0.001	0.012	<0.001	<0.001	<0.001
HSM	t = 0	1.7 d	3.5 b	1.8 c	2.5 c	8.0 d	1.9 b
	t = 1	76 a	14 a	40 a	59 a	59 a	8.6 a
	t = 2	17 b	4.7 b	11 b	21 b	21 b	2.2 b
	t = 3	7.4 c	4.1 b	12 b	13 b	15 c	2.0 b
	t = 4	7.6 c	4.0 b	17 b	12 b	13 c	1.9 b
	P	<0.001	<0.001	<0.001	<0.001	<0.001	<0.001
P[1]		<0.001	<0.001	<0.001	<0.001	<0.001	<0.001
LSD[1]		12.1	2.8	11.4	12.2	19.9	2.5

C = control (no smoke exposure); CM = control with misting; LS = low density smoke exposure; HS = high density smoke exposure; HSM = high density smoke exposure with misting. Values are means of three replicates (n = 3). Different letters (within columns, by treatment) indicate statistical significance (P = 0.05, one way ANOVA) amongst time points, i.e.: immediately prior to smoke exposure (t = 0); 1 hour after smoke exposure (t = 1); 1 day after smoke exposure (t = 2); 7 days after smoke exposure (t = 3); and 4 weeks after smoke exposure (t = 4) being maturity; ns = not significant. [1] P and LSD values for two way ANOVA of data by treatment and time.

Table S4. Mean intensity ratings for sensory attributes of control and smoke-affected wines.

Attribute	C	CM	LS	HS	HSM	P
fruit aroma	4.7 a	4.7 a	4.0 a	2.2 b	2.4 b	<0.0001
smoke aroma	1.8 c	2.1 bc	2.7 b	6.7 a	6.6 a	<0.0001
cold ash aroma	1.4 b	1.9 b	2.4 b	5.4 a	5.7 a	<0.0001
earthy aroma	2.6	2.5	2.9	3.1	2.9	ns
medicinal aroma	2.4 b	2.3 b	2.5 b	4.2 a	4.0 a	0.0001
burnt rubber aroma	1.3 b	1.2 b	1.4 b	4.0 a	3.7 a	<0.0001
fruit flavor	4.8 a	4.9 a	4.5 a	2.5 b	2.8 b	<0.0001
smoky flavor	1.7 b	1.9 b	2.2 b	6.3 a	6.1 a	<0.0001
medicinal flavor	1.8 b	1.6 b	1.7 b	3.6 a	3.8 a	<0.0001
ashy aftertaste	1.5 b	2.0 b	1.9 b	5.8 a	6.1 a	<0.0001
woody aftertaste	2.5 b	2.7 b	2.5 b	3.3 a	3.5 a	0.0025
metallic	2.1 b	2.1 b	2.0 b	3.5 a	3.4 a	0.0003
acidity	5.0	5.4	4.9	5.3	5.2	ns
hotness	3.3 bc	4.2 a	3.7 b	3.1 c	3.7 b	0.0002
bitterness	2.3 b	2.1 b	2.3 b	3.4 a	3.4 a	0.0004
drying	4.5 b	4.4 b	4.2 b	5.5 a	5.6 a	0.0025

C = control (no smoke exposure); CM = control with misting; LS = low density smoke exposure; HS = high density smoke exposure; HSM = high density smoke exposure with misting. Values are means for one wine per treatment presented to 50 judges. Different letters (within rows) indicate statistical significance (P = 0.05, one way ANOVA); ns = not significant.

Table S5. Basic composition of control and smoke-affected wines.

Measurement	C	CM	LS	HS	HSM	P
pH	3.7	3.7	3.7	3.6	3.6	ns
TA (g/L)	6.6 b	7.1 a	7.1 a	6.4 b	7.0 a	<0.001
alcohol (% abv)	11.5	12.9	11.9	10.6	12.5	ns
wine color density	6.7 bc	7.9 a	7.4 ab	6.5 c	8.2 a	0.005
wine color hue	0.89 a	0.82 b	0.83 b	0.87 a	0.82 b	0.002
total phenolics	137.7	137.0	137.2	138.1	141.0	ns

C = control (no smoke exposure); CM = control with misting; LS = low density smoke exposure; HS = high density smoke exposure; HSM = high density smoke exposure with misting. Values are means of three wine replicates. Different letters (within rows) indicate statistical significance (P = 0.05, one way ANOVA); ns = not significant.

Table S6. Viticultural measurements for control and smoke-affected grapevines.

Measurement	C	CM	LS	HS	HSM	P
TSS (° Brix)	20.8 ± 1.7%	22.3 ± 3.7%	19.6 ± 17.1%	19.6 ± 1.6%	21.7 ± 1.2%	ns
bunch number	67.3 ± 18.2%	58.0 ± 8.8%	59.0 ± 14.5%	71.7 ± 12.9%	56.3 ± 6.8%	ns
yield (kg)	6.7 ± 22.8%	5.4 ± 14.5%	5.4 ± 9.1%	6.9 ± 20.4%	4.7 ± 4.0%	ns
shoot number	45.3 ± 9.9%	42.3 ± 21.6%	42.3 ± 5.2%	51.0 ± 14.5%	37.3 ± 6.3%	ns
pruning weight (kg)	2.6 ± 9.4%	2.7 ± 25.9%	1.9 ± 1.9%	2.1 ± 17.8%	1.6 ± 5.9%	ns

C = control (no smoke exposure); CM = control with misting; LS = low density smoke exposure; HS = high density smoke exposure; HSM = high density smoke exposure with misting. Values are means of three replicates ± relative standard error. No statistical significance observed amongst treatments (P = 0.05, one way ANOVA); ns = not significant.

Table S7. Aroma and palate attributes used in sensory analysis of wines.

Attributes	Definition
Aroma	
fruit	intensity of the overall fruit aroma
smoke	perception of any type of smoke aroma, including smoked meat/bacon, toasty, charry, cigar-box, estery
cold ash	burnt aroma associate with ashes, including ashtray, tarry, campfire
earthy	any aroma associated with musty, dusty, wet-wood, barnyard, mushroom-like, dank, moldy, stagnant, stale
medicinal	aromatic characteristic of Band-Aids, disinfectant-like, including cleaning products, solvents, chemicals
burnt rubber	perception of burnt rubber-like aromas
Palate	
fruit	intensity of the overall fruit flavor
smoky	perception of smoke flavor, including bacon and smoked meat
ashy aftertaste	length of taste associated with residue of ashtray perceived in the mouth after expectorating, including coal ash, ashtray, tarry, acrid, campfire
woody aftertaste	length of taste associated with woody residue, includes wood, oak, pencil shavings
metallic	the ‘tinny’ flavor associated with metals
acidity	intensity of sour/acid taste
hotness	intensity of warmth/heat due to ethanol
bitterness	intensity of bitter taste, bitter aftertaste
drying	intensity of drying, puckering mouthfeel
